# Cerebral Tissue Oxygenation during Immediate Neonatal Transition and Resuscitation

**DOI:** 10.3389/fped.2017.00029

**Published:** 2017-02-23

**Authors:** Gerhard Pichler, Georg M. Schmölzer, Berndt Urlesberger

**Affiliations:** ^1^Division of Neonatology, Department of Pediatrics, Medical University, Graz, Austria; ^2^Centre for the Studies of Asphyxia and Resuscitation, Royal Alexandra Hospital, Edmonton, AB, Canada; ^3^Department of Pediatrics, University of Alberta, Edmonton, AB, Canada

**Keywords:** cerebral tissue oxygenation, monitoring, neonate, transition, resuscitation

## Abstract

This article provides a review of cerebral tissue oxygenation during immediate transition after birth in human neonates. Recommended routine monitoring, especially if resuscitation is needed, during this period includes arterial oxygen saturation and heart rate measured by pulse oximetry and electrocardiogram. However, there is increasing interest to monitor in addition with near-infrared spectroscopy (NIRS) the oxygenation of the brain. There is a different pattern of increase between cerebral tissue oxygenation and arterial oxygen saturation during the immediate transition, with cerebral tissue oxygenation reaching a plateau faster than arterial oxygen saturation. Differences can be explained, since cerebral tissue oxygenation is not only affected by arterial oxygen saturation but also by cerebral blood flow, hemoglobin content, and cerebral oxygen consumption. Normal values have already been established for different devices, gestational ages, and modes of delivery in neonates without any medical support. Cerebral hypoxia during immediate transition might cause brain damage. In preterm neonates with cerebral hemorrhage evolving in the first week after birth, the cerebral tissue oxygenation is already lower in the first minutes after birth compared to preterm neonates without cerebral hemorrhage. Using cerebral NIRS in combination with intervention guidelines has been shown to reduce the burden of cerebral hypoxia in preterm neonates. Cerebral tissue oxygenation during immediate transition seems to have an impact on outcome, whereby NIRS monitoring is feasible and has the advantage of continuous, non-invasive recording. The impact of NIRS monitoring and interventions on short- and long-term outcomes still need to be evaluated.

## Introduction

Transition from fetus to newborn is a complex physiological process. Monitoring this process to recognize perturbations is crucial but remains challenging. Initial assessment of neonates is usually based on visual inspection, palpation and/or auscultation, and response to stimuli. Until recently, decision to start and to guide medical support was based mainly on clinical assessment including muscle tone, reflexes, breathing, heart rate, and skin color. These parameters are summarized in the Apgar score, which was introduced by Apgar in 1952 (1). Her intention was to standardize clinical assessment and establish an objective prognostic tool. The Apgar score is now used routinely around the world due to the easy and fast application (2, 3). Several studies tried to establish associations between low score and short- and long-term outcome. However, there is a high interobserver variability especially in preterm neonates and neonates, who are in need of medical support (4–7).

Especially in preterm neonates, minimal handling and non-invasive monitoring is preferred. Recommended routine monitoring includes therefore pulse oximetry with measuring arterial oxygen saturation and heart rate on the one hand and electrocardiography (ECG) to monitor heart rate on the other hand (8–10).

Conflicting observations on heart rate were made when pulse oximetry was used in comparison to ECG (9, 11). Differences occur especially in the first minutes after birth (11). According to recent resuscitation guidelines, both methods for heart rate monitoring can be used during resuscitation and/or continued respiratory support (10). Concerning arterial oxygen saturation monitoring, there is an ongoing discussion what target saturation should be used, if the neonate needs respiratory support and oxygen supplementation. The resuscitation guidelines recommend the 25th percentile of published normal values for target saturation at different time points during the first minutes after birth (10).

However, recommended monitoring of vital signs does not include monitoring of the brain, one of the most vulnerable organs especially to hypoxia during transition immediately after birth. At the moment, only clinical assessment of neurological behavior and examination of muscle tone and reflexes of the newly born neonate is recommended to assess status of the brain. In the last decades, different methods of monitoring during the immediate transition after birth have been tried and described.

First, there is Doppler sonography with the advantage of non-invasive monitoring the cerebral perfusion of different regions of the brain. However, this method is limited since it is affected by movement of the infant and parameters cannot be acquired continuously. Furthermore, it is technically difficult to perform during immediate transition (12).

Second, amplitude integrated electroencephalography enables measurement of cerebral activity with the advantage of continuous, non-invasive recordings and is also limited by the technical difficulties of sampling and interpretation due to artifacts (12).

Third, there is the monitoring of cerebral tissue oxygenation by means of near-infrared spectroscopy (NIRS)—a method that enables to monitor both, changes in cerebral oxygenation and perfusion. There is an increasing interest to apply NIRS during immediate transition after birth. This article provides a review of the literature (Table 1) on cerebral regional oxygenation measured with NIRS during the immediate transition after birth in human neonates.

**Table 1:** **Cerebral tissue oxygenation immediately after birth in human neonates**.

Reference	Term/preterm	Device	Study type
Peebles et al. (13)	Term	/	Case report
Isobe et al. (14)	Term	IMUC 7000	Observational
Isobe et al. (15)	Term	IMUC 7000	Observational
Fauchère et al. (16)	Term	NIRO 300	Observational
Urlesberger et al. (17)	Term	INVOS 5100	Observational
Urlesberger et al. (22)	Term	INVOS 5100	Observational
Fuchs et al. (19)	Preterm	FORESIGHT	Observational
Kratky et al. (18)	Term	INVOS 5100	Observational
Noori et al. (26)	Term	INVOS	Observational
Fuchs et al. (20)	Preterm	FORESIGHT	Observational
Binder et al. (30)	Preterm	INVOS 5100	Observational
Urlesberger et al. (22)	Term	INVOS 5100	Observational
Pichler et al. (39)	Term	INVOS 5100	Observational
Pichler et al. (35)	Term/preterm	INVOS 5100	Observational
Almaazmi et al. (24)	Term	FORESIGHT	Observational
Hessel et al. (37)	Term	INVOS/FORESIGHT	Observational
Karen et al. (25)	Term	NIRO 300	Observational
Li et al. (21)	Preterm	INVOS 5100	Case report
Schwaberger et al. (31)	Preterm	INVOS 5100	Observational
Baik et al. (36)	Term	NIRO 200 NX	Observational
Baik et al. (41)	Preterm	INVOS 5100	Observational
Kenosi et al. (38)	Preterm	INVOS 5100	Observational
Montaldo et al. (23)	Term	Nonin	Observational
Pocivalnik et al. (34)	Term	INVOS 5100	Observational
Schwaberger et al. (29)	Preterm	NIRO 200 NX	RCT
Schwaberger et al. (33)	Term	NIRO 200 NX	Observational
Baik et al. (28)	Preterm	INVOS 5100	Observational
Pichler et al. (42)	Preterm	INVOS 5100	RCT
Pichler et al. (32)	Preterm	INVOS 5100	Observational
Tamussino et al. (40)	Term	INVOS 5100	Observational

## Discussion

Peebles et al. described measurements of cerebral hemodynamics using NIRS during immediate transition after birth of a healthy term neonate in 1992 for the first time (13). The NIRS sensors were applied to the head of the neonate during labor before birth. They were able to demonstrate rapid changes in cerebral oxygenation during fetal to neonatal transition especially with onset of respiration. Unfortunately, it took a decade until first studies using cerebral NIRS immediately after birth were reported (14, 15), and another 10 years until studies were performed to monitor cerebral tissue oxygenation and hemodynamics using NIRS immediately after birth.

In the last decade, several studies have demonstrated feasibility of cerebral NIRS during immediate transition in healthy term neonates (13–18), preterm neonates (19, 20), and even during resuscitation with chest compressions (21).

Cerebral tissue oxygenation can be obtained quickly within 2 min after birth, thus enabling continuous non-invasive monitoring immediately after birth. Several studies have demonstrated that cerebral oxygenation changes differently within the first minutes after birth when compared to arterial oxygen saturation, heart rate, or even peripheral tissue oxygenation (17, 22). Cerebral tissue oxygenation reaches a plateau faster compared to arterial oxygen saturation, thus indicating that there might be a preferential oxygen delivery to the brain in the first minutes after birth (17, 23). Furthermore, changes in cerebral tissue oxygenation and arterial oxygen saturation depend on mode of delivery (15, 22, 24, 25). While arterial oxygen saturation and heart rate changes differently after cesarean section and vaginal delivery, cerebral tissue oxygenation shows similar changes after both modes of delivery.

Cerebral tissue oxygenation is affected by arterial oxygen saturation and also by cerebral blood flow, hemoglobin content, and cerebral oxygen consumption, which may explain the differences between cerebral tissue oxygenation and arterial oxygen saturation. Cerebral blood flow depends on cardiac output, vascular resistance, and especially during immediate transition on open shunts. Noori et al. demonstrated that ductal shunting rapidly changes from balanced to left to right after birth, with a responsive increase in left ventricular stroke volume (26). Cerebral oxygen saturation increases as arterial oxygen saturation rises during the first minutes. Urlesberger et al. demonstrated that during postnatal transition term infants with left-to-right shunt via the ductus arteriosus have significantly higher cerebral tissue oxygenation values compared to infants without shunts (27). Additionally, shunting from the “foramen ovale” shunting might also have an influence on cerebral perfusion thus cerebral oxygenation. The increasing sum of ductus arteriosus and “foramen ovale” diameters were correlated negatively with cerebral oxygen saturation (28). These data suggest that all shunts have an influence on cerebral tissue oxygenation during immediate neonatal transition. In healthy newborns immediately after birth, there is a significant decrease in cerebral blood volume (29). This is most probably caused by cerebral vasoconstriction due to the steep increase in arterial oxygen tension and may be regarded as a physiological process. This suggests that a reduction in cerebral blood flow is likely due to (i) increase in arterial oxygen content, (ii) shunting via the ductus arteriosus and/or foramen ovale, or (iii) very often a combination of these mechanisms.

Any disturbances during immediate transition and necessary interventions during resuscitation might therefore influence transitional physiological cerebral oxygenation and hemodynamics. Neonates in need of respiratory support have compromised cerebral tissue oxygenation compared to neonates with undisturbed transition. Differences between neonates needing respiratory support and neonates with uncomplicated transition after birth have been observed for cerebral tissue oxygenation as well as for cerebral tissue oxygen extraction (calculated out of arterial oxygen saturation and cerebral tissue oxygen saturation). These observations indicate that decreased cerebral tissue oxygenation in neonates with respiratory support might be due to lower arterial oxygen saturation levels as well as compromised perfusion (30, 31). Delayed cord clamping is an intervention recommended by resuscitation guidelines. However, delayed cord clamping causes lower initial cerebral tissue oxygenation in spontaneous breathing preterm neonates when compared to preterm neonates with early cord clamping (32). This initial lower cerebral oxygenation might be explained by inadequate lung aeration due to delayed adequate respiratory support during delayed cord clamping. Sustained lung inflation, which remains a controversial intervention, might affect cerebral blood volume in preterm neonates during the first minutes after birth (33). Neonates receiving sustained lung inflation had no physiological decrease in cerebral blood volume during the first minutes after birth compared to neonates with conventional respiratory support. The authors of this study hypothesized that after sustained lung inflation an impaired venous return to the heart led to cerebral venous stasis and the potential hazardous differences in cerebral blood volume behavior (33). In term newborns, oropharyngeal suctioning, often routinely performed, did not compromise cerebral tissue oxygenation (34).

Reference ranges have been established in healthy term neonates after vaginal delivery and cesarean section as well as in healthy late preterm neonates after cesarean section with the INVOS 5100 (35). Median (10th–90th percentiles) cerebral regional oxygen saturation measured with INVOS 5100 was 41% (23–64) at 2 min, 68% (45–85) at 5 min, 79% (65–90) at 10 min, and 77% (63–89) at 15 min after birth. Reference ranges have also been established with the NIRO 200 in healthy term neonates (36). Median (10th–90th percentiles) “cerebral tissue oxygenation index” measured with NIRO 200 NX was 56% (39–75) at 2 min, 66% (50–78) at 5 min, 75% (62–85) at 10 min, and 73% (61–84) at 15 min after birth. However, there were differences between these two devices. Differences between NIRS devices are well known, and they are most probably related to differences in algorithms used to calculate values. A comparison of INVOS 5100 and FORESIGHT also reported differences between the two devices (37). The INVOS and FORESIGHT cerebral oxygenation estimates showed oxygenation level-dependent difference during birth transition. Published percentile should therefore only be used as reference range, when the same device, with which a percentile has been established, is used.

Reference ranges have also been published for very low birth weight preterm neonates (19, 20). However, especially in preterm neonates in need of medical support, reference ranges have to be interpreted with caution since percentiles depend on medical support given (e.g., amount of supplemental oxygen, respiratory support). For example, infants given >0.3 FiO_2_ are described to have more cerebral hypoxia than infants requiring <0.3 FiO_2_ but there is no difference in the degree of cerebral hyperoxia, both in the delivery suite and the NICU (38).

Both, hypoxia and bradycardia, are common events during immediate transition in preterm neonates. In particular, cerebral hypoxia–ischemia may cause perinatal brain injury, which is a major cause of mortality and long-term neurodevelopmental impairment in preterm neonates. Cerebral hypoxia during immediate transition might be associated with alteration in cerebral activity (39), whereby neonates with initially low cerebral activity at birth showed low cerebral tissue oxygenation (<10th percentile), but increased cerebral oxygen extraction (>90th percentile) (40).

Further, preterm neonates requiring respiratory support could have arterial oxygen saturation within normal ranges; however, cerebral tissue oxygenation might remain hypoxic. In a two-center prospective observational cohort study, effects of cerebral tissue oxygenation on perinatal brain injury in preterm infants <32 weeks of gestational age during the immediate neonatal transition (15 min) were examined (41). Neonates, who developed intraventricular hemorrhage during the first week after birth, showed significantly lower cerebral tissue oxygenation already within the first 15 min after birth compared to neonates without intraventricular hemorrhage. However, no difference between groups in arterial oxygen saturation or heart rate was observed (41).

Furthermore, a prospective randomized controlled pilot study compared supplemental oxygen delivery either via cerebral tissue oxygenation monitoring in addition to arterial oxygen saturation (NIRS-visible group) to arterial oxygen saturation monitoring alone (NIRS-not-visible group) in preterm neonates <34 + 0 weeks of gestation (42). The primary outcomes were burden of cerebral hypoxia (<10th percentile) or hyperoxia (>90th percentile) measured in %minutes cerebral tissue oxygenation during the first 15 min after birth. Overall, in the NIRS-visible-group burden of cerebral hypoxia in %minutes was halved, whereby relative reduction was 55.4%. There was a trend to lower mortality and/or cerebral injury in NIRS-visible-group compared to control group (13 vs. 20%) (42).

In the last decade, several studies have addressed cerebral tissue oxygenation during the immediate transition in term and preterm neonates and have proven feasibility of measurement. Limitation of NIRS includes differences between devices, which should be considered when normative data or data of different studies are compared. Penetration depth of the mainly used devices in neonates is 15–20 mm (half of distance from light emitter to sensor); thus, NIRS mainly measures oxygenation in brain cortex and not the brain stem. Regardless, NIRS provides the advantage of continuous non-invasive measurement of cerebral tissue oxygenation with fast acquisition time (within 2 min after birth) and potentially has a role in guiding medical support and resuscitation in neonates with compromised transition. Current available data demonstrate that cerebral hypoxia can be reduced by monitoring cerebral tissue oxygenation with NIRS in combination with intervention guidelines. The next step would be a larger randomized controlled trial to investigate the effect of cerebral NIRS monitoring in combination with intervention guidelines on short- and long-term outcome in preterm neonates, which is currently underway.

## Author Contributions

Conception and design; critical revision of the article for important intellectual content; final approval of the article: GP, GS, and BU. Drafting of the article: GP.

## Conflict of Interest Statement

The authors declare that the research was conducted in the absence of any commercial or financial relationships that could be construed as a potential conflict of interest.
